# Hepatitis E Virus Infection in Macaca Mulatta

**DOI:** 10.5812/kowsar.1735143x.783

**Published:** 2011-10-01

**Authors:** Elham Shirvani Dastgerdi, Samad Amini-Bavil-Olyaee

**Affiliations:** 1 Medical Clinic III, RWTH-University Hospital Aachen, Pauwelsstrasse, Aachen, Germany; 2 Biotechnology Department, Pasteur Institute of Iran, Tehran, IR Iran

**Keywords:** Hepatitis E Virus, Macaca Mulatta, Genotype

Dear Editor,

One of the ﬁve known hepatitis viruses that can infect humans is hepatitis E virus (HEV). The ﬁrst documented infection that was caused by HEV occurred in 1955 during an outbreak in New Delhi, India [1]. HEV is a small nonenveloped particle that belongs to the family Hepeviridae and the genus Hepevirus. Its single-stranded RNA genome has approximately 7200 base pairs [2]. By genomic sequence analysis, there are four genotypes [1][2][3][4] and at least 24 subgenotypes (1a-1e, 2a-2b, 3a-3j, and 4a-4g) of HEV in humans and other mammals [4]. Avian isolates of HEV were initially proposed to constitute the ﬁfth genotype of HEV; but, due to their shorter genome and low homology with mammalian isolates, they have been categorized as a member of a separate genus [3]. HEV genotypes 1 and 2 are limited to humans, and genotypes 3 and 4 are common between humans and other mammalian species [5]. Domestic animals, especially pigs, are the principal animal reservoirs for HEV [6], as are, to a lesser extent, boar, deer, rabbits, and rats [7][8]. In a study in China, a serum positivity test for anti-HEV antibodies was performed for farm animals. The highest positive rates of serum anti-HEV were detected in swine (81.17%), rabbit (54.62%), and cattle (25.29%) [9].

Several animal species, such as chimpanzees; rhesus and cynomolgus macaques; and, more recently, pigs, rabbits, and chickens, have been used as animal models to study different aspects of HEV infection and for vaccine trials [10]. Despite supporting HEV replication and producing antibodies against the virus, there are insufficient studies on the capacity of monkeys to serve as natural reservoirs for the virus. Huang et al. in a study on HEV seroepidemiology in China, found anti-HEV IgG and IgM in the serum of rhesus monkeys (Macaca mulatta) but not HEV RNA in their stool [11]. Regarding the possibility of cross-species infection between wild animals or between human and animals, such as on farms, knowledge of the seroepidemiology of the virus and its natural reservoirs seems to be essential. Workers at pig farms and slaughterhouse workers have twice the level of anti-HEV compared with the general population [9]. This may indicate the role of zoonotic infection by HEV. This is important, especially in xenotransplantations [12]. Another study showed that in an area with both swine and Macaca mulatta, antibodies against HEV were present in both groups. However, despite the detection of swine HEV genotype 4 RNA in stool specimens, the same assay was negative for Macaca mulatta. Swine HEV can infect nonhuman primates; cross-species transmission of the virusis possible [12], although positive antibody detection can also be due to the existence of HEV-like proteins [11][13] or incomplete replication of swine HEV in Macaca mulatta. Genomic changes (mutation or recombination) in the viral genome can lead to new genotype or subgenotype generations, rendering them undetectable by existing molecular approaches. Although the role of Macaca mulatta as a natural reservoir for HEV has been failed [11], its seropositivity for viral antibodies against HEV should not be underestimated. Thus, more studies are necessary to define HEV seroepidemiology in nonhuman hosts.
